# Function value of basement membrane-related genes in odontogenic keratocyst by bioinformatics analysis

**DOI:** 10.3389/fonc.2025.1658125

**Published:** 2025-09-12

**Authors:** Jing-Rui Yi, Nian-Nian Zhong, Xuan-Hao Liu, Zheng-Rui Zhu, Yi-Jia-Ning Zhang, Bing Liu, Qi-Wen Man

**Affiliations:** ^1^ State Key Laboratory of Oral & Maxillofacial Reconstruction and Regeneration, Key Laboratory of Oral Biomedicine Ministry of Education, Hubei Key Laboratory of Stomatology, School & Hospital of Stomatology, Wuhan University, Wuhan, China; ^2^ Department of Oral & Maxillofacial - Head Neck Oncology, School and Hospital of Stomatology, Wuhan University, Wuhan, China

**Keywords:** odontogenic keratocyst, basement membrane, SPARC, RNA sequencing, single-cell RNA sequencing

## Abstract

**Background:**

Odontogenic keratocyst (OKC) is an aggressive jaw lesion characterized by high recurrence rates. Disruption of the basement membrane (BM) may contribute to its pathogenesis, through the underlying molecular mechanisms remain incompletely understood.

**Methods:**

Transcriptomic data from 12 non-syndromic OKC and 4 normal oral mucosa (NOM) samples (GSE38494) were analyzed to identify differentially expressed BM-related genes (BM DEGs). Bioinformatics approaches included differential expression analysis, functional enrichment (Gene Ontology, Kyoto Encyclopedia of Genes and Genomes, Gene Set Enrichment Analysis), protein-protein interaction (PPI) network construction, immune infiltration assessment, and validation via single-cell RNA-seq (scRNA-seq; GSE176351). Key findings were confirmed immunohistochemistry and by immunofluorescence in clinical specimens.

**Results:**

A total of 65 BM DEGs were identified, with secreted protein acidic and cysteine rich (SPARC) being the most significantly upregulated gene (P < 0.01). PPI and correlation analyses established SPARC as a hub gene, showing significant correlation with recognized OKC markers (PTCH1, GLI1, GLI2, KRT19; P < 0.05). ScRNA-seq localized elevated SPARC expression predominantly to stromal fibroblasts. Immunohistochemistry and immunofluorescence confirmed significantly higher stromal SPARC expression in OKC versus NOM (P = 0.001). SPARC levels correlated with altered immune infiltration profiles, showing positive association with effector memory CD4+ T cells and negative association with memory B cells. Transcription factor and microRNA regulatory networks for SPARC were delineated.

**Conclusions:**

This study establishes dysregulation f BMGs—particularly stromal fibroblast-specific SPARC overexpression—as a contributor to OKC pathogenesis. SPARC interacts with key OKC pathways (Hedgehog, NOTCH) and modulates the immune microenvironment. These findings provide foundational insights into OKC aggressiveness and propose SPARC as a potential therapeutic target.

## 1 Introduction

Odontogenic keratocyst (OKC) is locally aggressive jaw lesions characterized by a high recurrence rate (1). Previously classified as keratocystic odontogenic tumors (KCOTs), OKCs were re-designated by the World Health Organization in 2017 (2). OKCs can be divided into syndromic OKC (S-OKCs), which are associated with genetic disorders such as Gorlin-Goltz syndrome (nevoid basal cell carcinoma syndrome, NBCCS), and non-syndromic OKCs (NS-OKCs), which occur sporadically without systemic manifestations. Compared to other odontogenic cysts—such as dentigerous cysts (DCs) and radicular cysts (RCs)—OKCs exhibit more aggressive behavior and a higher risk of recurrence (3, 4).

Histologically, OKCs are characterized by a parakeratinized stratified squamous epithelium that is thin and fragile, making it prone to fragmentation during surgical removal. This fragility increases the likelihood of residual epithelial remnants, which, together with the satellite (daughter) cysts—whose exact origin remains unclear—contribute to the high recurrence rate of OKCs. These features pose significant challenges for complete surgical excision (5, 6). The Hedgehog signaling pathway and mutations in *PTCH1* are widely recognized as major contributors to OKC pathogenesis (7). Recent studies have also investigated the role of immune cell infiltration and oxidative stress–related genes in OKCs (8, 9). In addition, network pharmacology approaches have been applied to identify potential therapeutic targets (10).

The basement membrane (BM) is a specialized extracellular matrix (ECM) that separates epithelial cells from the underlying stroma. It is composed of structural proteins such as laminins and type IV collagen, as well as proteoglycans like perlecan, and plays a critical role in maintaining epithelial architecture, guiding tissue development, and preserving homeostasis (11, 12). BM disruption has been implicated in several oral mucosal diseases, including mucous membrane pemphigoid and epidermolysis bullosa (13). In cancer biology, alterations in BM stiffness and protein composition are known to modulate tumor cell invasion and metastasis, with changes in laminin and type IV collagen correlating with disease progression (14).

In OKCs, aberrant BM patterns may contribute to their aggressive behavior. Poomsawat et al. reported discontinuous expression of type IV collagen and fibronectin in OKC specimens (11). Additionally, differences in BM component expression, such as laminin and fibronectin, have been observed between S-OKCs and NS-OKCs (15). Collagen type I, detected in the BM of OKCs, has been shown to enhance IL - 1–mediated activation of MMP - 2, promoting osteoclastic activity and cyst expansion (16), suggesting a potential role in local invasiveness. However, the molecular mechanisms by which BM components contribute to OKC progression remain largely unclear.

Bioinformatics has become an essential tool in OKC research, enabling large-scale analyses in genomics, transcriptomics, and proteomics (17). To explore the relationship between BM components and OKC, we utilized the bmBASE database (https://bmbase.manchester.ac.uk), which catalogs 160 BM matrix proteins and 62 cell surface interactor (CSI) genes with conserved BM localization across species (18). This database integrates structural domain information, antibody data, and subcellular localization to support functional and comparative studies of BM-related genes (BMGs). Transcriptomic data used in our study were obtained from the Gene Expression Omnibus (GEO) database (https://www.ncbi.nlm.nih.gov/geo/), encompassing gene expression profiles from ameloblastoma (AM), OKC, and normal oral mucosa (NOM) (19).

In this study, we systematically analyzed BMG expression in OKC and NOM using publicly available transcriptomic datasets. By identifying differentially expressed BMGs and characterizing key candidates, we aimed to elucidate their potential roles in OKC pathogenesis. We further validated the expression and function of a representative gene—secreted protein acid and rich in cysteine (SPARC), providing mechanistic insights and a theoretical foundation for future targeted therapeutic strategies.

## 2 Materials and methods

### 2.1 Specimen collection

OKC tissue samples were collected from patients undergoing cyst curettage at the Department of Oral & Maxillofacial - Head Neck Oncology, Hospital of Stomatology, Wuhan University. NOM tissue was obtained from patients undergoing cleft lip and palate repair surgeries. All tissues were initially fixed in 4% paraformaldehyde, embedded in paraffin, and sectioned for further analysis. The procedures adhered to the guidelines of the National Institutes of Health for the use of human tissues. Ethical approval was granted by the Ethics Committee of the Hospital of Stomatology, Wuhan University (IRB-ID: 2020A95), and informed consent was obtained from all participants.

### 2.2 Identification and functional enrichment of BM DEGs

RNA-seq datasets were obtained from the GEO, including 12 NS-OKC samples and 4 NOM samples (GSE38494). Based on the study by Jayadev et al., 222 BMGs were defined (18). The intersection between differentially expressed genes (DEGs) and BMGs—differentially expressed BMGs (BM DEGs)—was identified using the R package “ggvenn”. Gene expression was analyzed using the “limma” package, and genes with |log_2_FC| > 1 and P < 0.05 were considered statistically significant. Visualizations included a heatmap generated with “pheatmap”, boxplots and volcano plots using “ggplot2”, and correlation plots using “corrplot”.

Functional enrichment analysis was conducted using Gene Ontology (GO, http://www.geneontology.org) and Kyoto Encyclopedia of Genes and Genomes (KEGG, http://www.genome.jp/kegg) databases and visualized via “ggplot2”. Gene Set Enrichment Analysis (GSEA) was performed using the ranked gene list (ordered by log_2_ fold-change from “limma” analysis) against the Molecular Signatures Database (MSigDB, www.gseamsigdb.org) Hallmark gene sets, implemented via the R package “clusterProfiler”. Significance was defined by thresholds of |NES| > 1.5, nominal P < 0.05, and FDR < 0.25 (1,000 permutations).

### 2.3 Generation of PPI network

The STRING database (https://string-db.org/) was used to construct a protein-protein interaction (PPI) network of BM DEGs, with the minimum confidence score set at 0.7 (high confidence). Cytoscape software was used to modify and visualize the final PPI network.

### 2.4 Acquisition and processing of OKC-related genes and validation datasets

Twenty-three genes most closely associated with OKC (ranked by correlation score) were obtained from the GeneCards database (www.genecards.org) for subsequent correlation and expression analysis. Additionally, two datasets, GSE228393 and GSE186489, were retrieved from GEO. Three OKC samples were selected from GSE186489 and three NOM samples from GSE228393. The expression matrices of these datasets were merged, and batch effects were corrected using the “sva” package to construct a validation dataset. Receiver operating characteristic (ROC) curves were plotted using the “pROC” package.

### 2.5 GSVA

Gene set variation analysis (GSVA) is an unsupervised, non-parametric method used to estimate variation of gene set enrichment across samples in microarray and RNA-seq data. GO sets were downloaded from the MSigDB. Samples were grouped into high and low SPARC expression groups based on the median expression value. Differential pathway analysis was performed using the “limma” package and visualized accordingly.

### 2.6 ScRNA-seq analysis

The GSE176351 dataset, containing single-cell RNA-seq (scRNA-seq) data from two primary and one recurrent OKC sample, was downloaded from GEO. Quality control was performed, and the datasets were integrated using the “harmony” package. Cell clustering and visualization were performed using the “seurat” package.

### 2.7 Construction of SPARC TF-miRNA regulatory network

The transcription factor (TF)-microRNA (miRNA) regulatory network for SPARC was constructed using the NetworkAnalyst (www.networkanalyst.ca). TF data were sourced from the ChIP-X database (20), and miRNA data were obtained from miRTarBase v8.0 (21). The predicted results were modified and visualized using Cytoscape software.

### 2.8 Immunoinfiltration analysis of SPARC

A gene set of 782 genes was used to assess the abundance of 28 tumor-infiltrating immune cell (TIIC) types in OKC tissue samples, based on MSigDB (https://www.gsea-msigdb.org/gsea/msigdb/index.jsp). Datasets GSE38494, GSE180706, and GSE186489 were merged, and batch effects were removed using the “sva” package. Boxplots of TIIC abundance were generated, and the correlation between SPARC expression and TIICs was analyzed and visualized.

### 2.9 Immunohistochemistry

Tissues were embedded in paraffin and sectioned at 4 μm thickness. Sections we baked at 60°C, followed by xylene (#1330-20-7, Komio) dewaxing and graded ethanol hydration. After phosphate-buffered saline (PBS) washing, antigen retrieval was performed using a sodium citrate buffer (#CR2202096, Servicebio) and microwave heating. Following cooling, endogenous peroxidase was blocked, and serum blocking solution (rabbit, #KIT-9707, Fuzhou Maixin) was applied for 20 minutes at 37°C. Sections were incubated overnight at 4°C with SPARC antibody (1:100, rabbit, #A14494, ABclonal), followed by secondary antibody (biotin-labeled anti-rabbit IgG polymer) and streptavidin-peroxidase complex (#KIT-9707, Fuzhou Maixin), each incubated at 37°C for 20 minutes. 3,3’-Diaminobenzidine (DAB) (#2210270031L, Fuzhou Maixin) and hematoxylin (#CR2109027, Servicebio) were used for color development. Slides were dehydrated, cleared, sealed with neutral mounting medium (#96949-21-2, Shanghai Test), and scanned using the Aperio ScanScope CS system (Sausalito, CA, USA).

### 2.10 Immunofluorescence

Tissue preparation followed the same protocol as for immunohistochemistry. After dewaxing and antigen retrieval, endogenous peroxidase and serum blocking were performed as described. SPARC antibody (1:100, rabbit, #A14494, ABclonal) was applied overnight at 4 °C. After PBS washing, fluorescent secondary antibody (AlexaFluor 488-conjugated goat anti-rabbit IgG; #A23220, Abbkine) was added and incubated at 37 °C for 1 hour in the dark. Nuclei were stained with 4’,6-diamino-2-phenyllindol (DAPI) (#ANT165, AntGene), and slides were sealed with antifade mounting medium (#ANT061, AntGene). Images were captured using a fluorescence microscope (Olympus IX83, Tokyo, Japan) with a 20× objective lens. SPARC was visualized in the green channel, and DAPI was detected in the blue channel.

### 2.11 Data analysis

All bioinformatics analyses were conducted using R software (version 4.2.3). Group comparisons were performed using the Wilcoxon rank-sum test. Spearman correlation was used for correlation analyses. A P value < 0.05 was considered statistically significant. GraphPad Prism 7.0 (GraphPad software, San Diego, CA, USA) was used for additional statistical analyses, including t-tests for comparing group means.

## 3 Results

### 3.1 Differential expression of BMGs in OKC and NOM tissues

**Figure 1:**
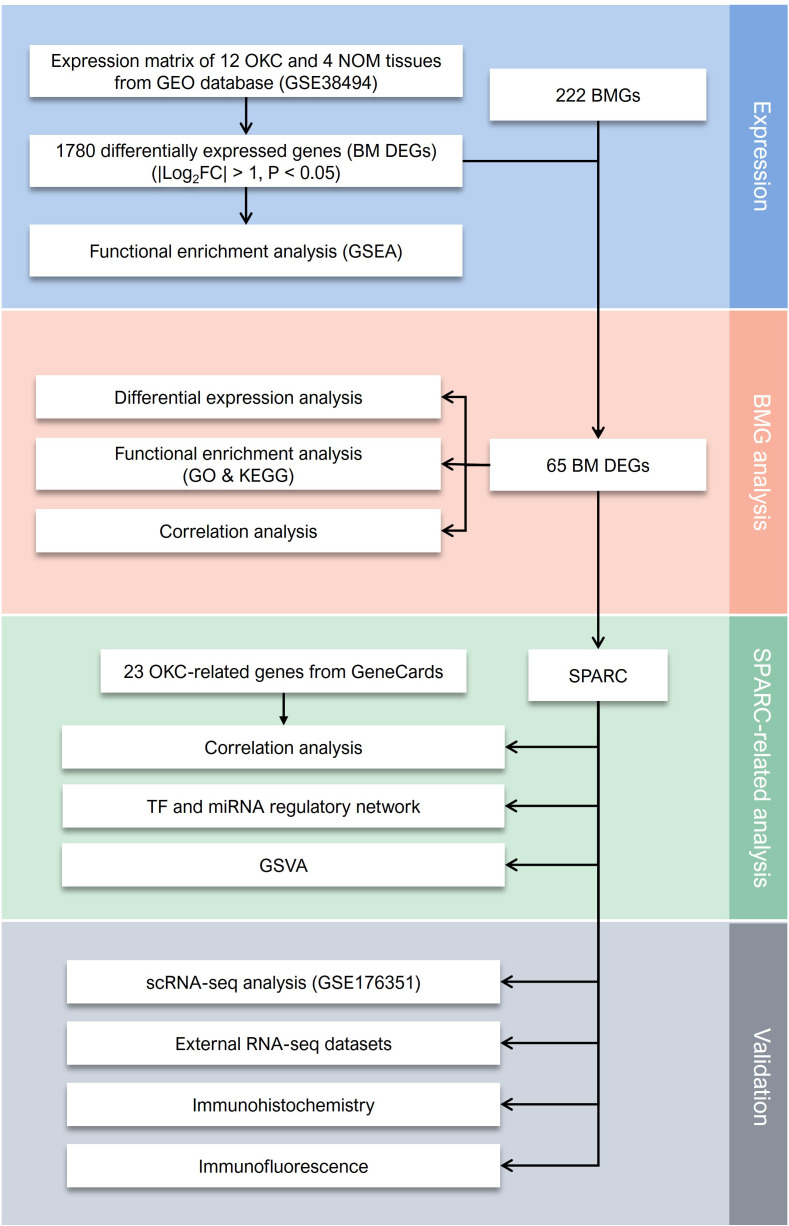
The flowchart of present study. BM DEGs, differentially expressed basement membrane-related genes; BMGs, basement membrane-related genes; GEO, Gene Expression Omnibus; GO, Gene Ontology; GSEA, Gene Set Enrichment Analysis; GSVA, Gene Set Variation Analysis; KEGG, Kyoto Encyclopedia of Genes and Genomes; NOM, normal oral mucosa; OKC, odontogenic keratocyst; RNA-seq, RNA sequencing; scRNA-seq, single-cell RNA sequencing; SPARC, secreted protein acidic and cysteine rich.

**Figure 2:**
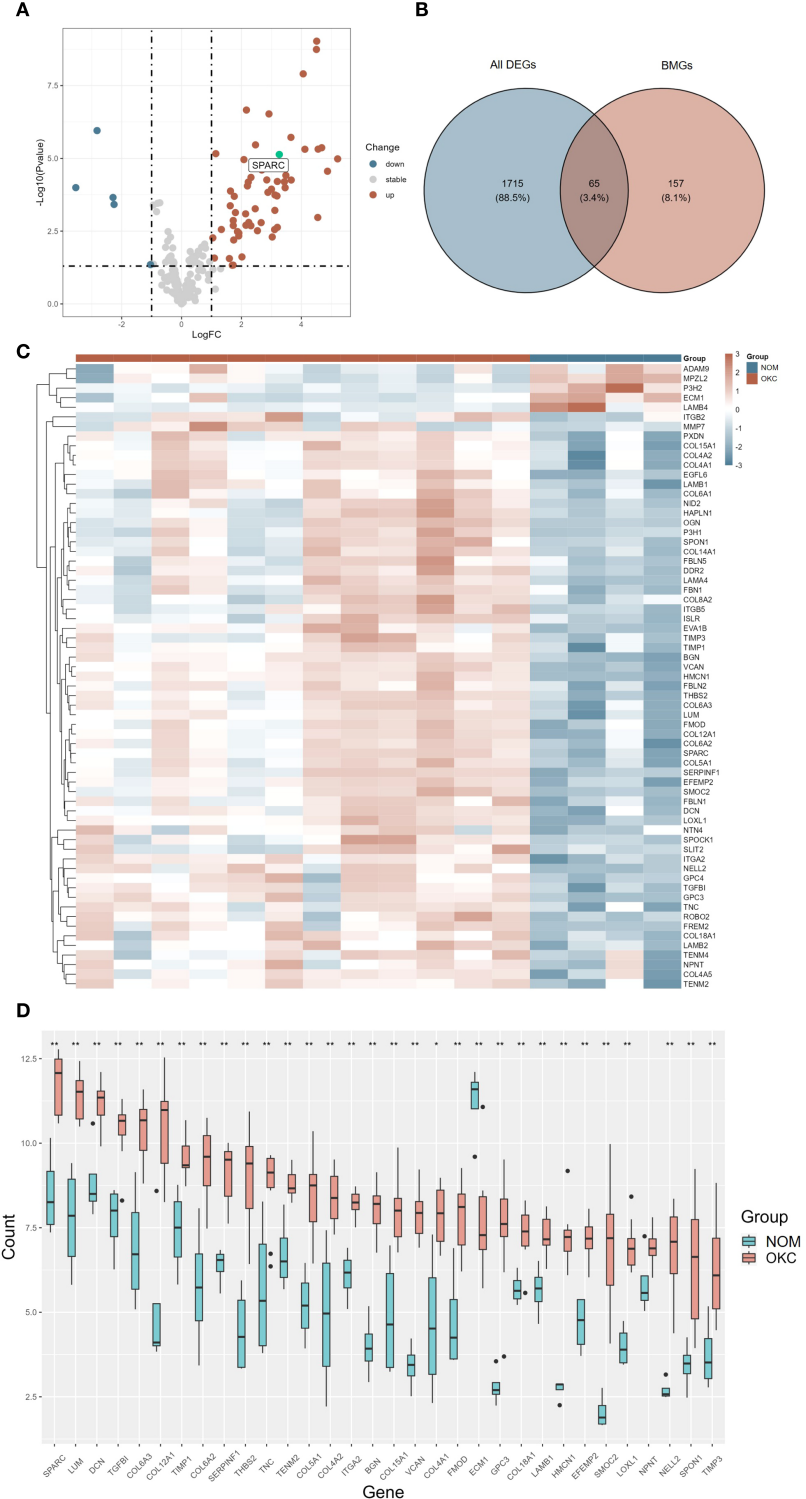
Differential expression analysis of BMGs in OKC and NOM samples. **(A)** Volcano plot **(A, B)** Venn diagram, and **(C)** heatmap illustrating DEGs. **(D)** Box plot showing the expression levels of the top 32 differentially expressed BMGs in NOM and OKC. Blank indicates not significant; *P < 0.05; **P < 0.01. BMGs, basement membrane-related genes; DEGs, differentially expressed genes; NOM, normal oral mucosa; OKC, odontogenic keratocyst.

Gene expression data from 12 NS-OKC samples and 4 NOM samples GSE38494 were obtained from the GEO database and used as the experimental dataset. An overview of the analysis workflow is presented in **Figure 1**. A total of 222 BMGs were retrieved from the bmBASE database. Using the “limma” package, differential expression analysis was conducted, and the results were visualized via a volcano plot (**Figure 2A**, **Supplementary Table S1**). Applying the thresholds of |log2FC| > 1 and P < 0.05, 1,780 DEGs were identified. Intersecting these with the BMGs yielded 65 BM DEGs (**Figure 2B**, **Supplementary Table S2**). Heatmaps and boxplots were generated to visualize the expression differences between groups (**Figures 2C, D**, **Supplementary Figure S1**, **Supplementary Table S3**). Among these, SPARC showed the highest expression level in OKC tissues, with a statistically significant difference (P < 0.01).

### 3.2 Functional enrichment and correlation analysis of BM DEGs

GO and KEGG enrichment analyses were conducted to explore the biological significance of the 65 BM DEGs (**Supplementary Figures S2A, B**, **Supplementary Tables S4**, **S5**). GO analysis revealed significant enrichment in biological processes such as “extracellular matrix organization”, “extracellular structure organization”, and “external encapsulating structure organization”; cellular component including “collagen-containing extracellular matrix” and “basement membrane”; and molecular functions such as “extracellular matrix structural constituent” and “extracellular matrix binding”. KEGG pathway analysis indicated that BM DEGs were significantly enriched in “ECM-receptor interaction”, “protein digestion and absorption”, “focal adhesion”, and “PI3K-Akt signaling pathway”. GSEA of all DEGs identified the top five upregulated (**Supplementary Figures S3A–E**) and top five downregulated (**Supplementary Figures S3F–J**) pathways (**Supplementary Table S6**).

**Figure 3:**
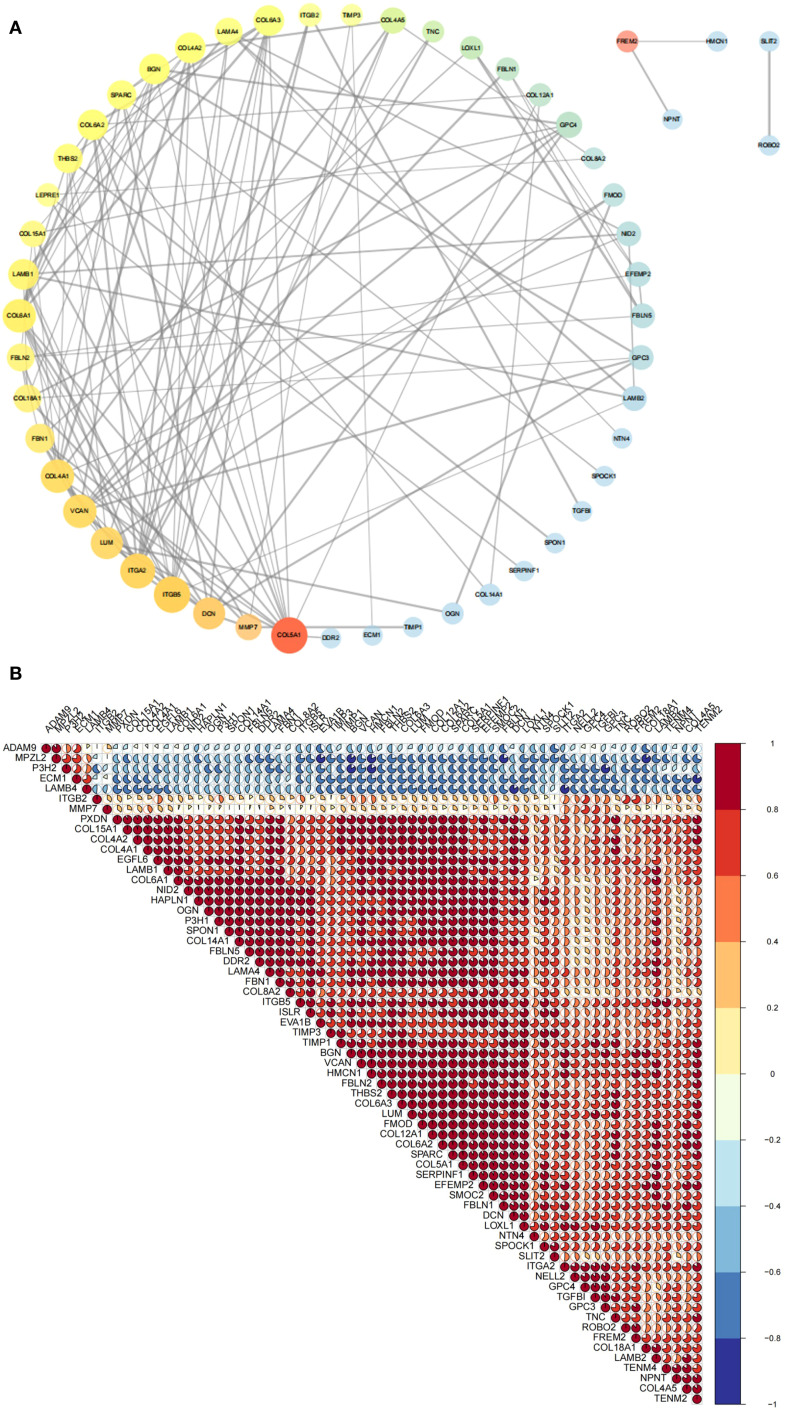
Correlation analysis of BM DEGs at the protein and RNA levels. **(A)** PPI network depicting interactions among 65 BM DEGs. Node size and transparency reflect degree centrality (larger and more opaque nodes indicate greater protein interaction). Node color, ranging from red to blue, represents betweenness centrality (warmer colors indicate higher centrality and thus greater importance in the network). Edge thickness corresponds to the combined interaction score (thicker lines denote stronger interaction credibility. **(B)** Pie chart displaying RNA-level correlations among 65 BM DEGs. Pie size and color indicate correlation magnitude and direction (warmer color reflect stronger positive correlations; cooler colors indicate stronger negative correlations). BM DEGs, differentially expressed basement membrane-related genes; PPI, Protein-protein interaction.

PPI analysis was performed using the STRING database with a confidence score threshold of ≥ 0.7, resulting in a PPI network that highlighted key interactions among BM DEGs (**Figure 3A**, **Supplementary Table S7**). Additionally, correlation analysis based on gene expression profiles was performed, and the results were visualized as a correlation plot (**Figure 3B**, **Supplementary Table S8**). Both the PPI and correlation analyses identified SPARC as a central hub gene with strong associations to multiple other BMGs.

### 3.3 Correlation between SPARC and OKC-related genes, and GSVA

**Figure 4:**
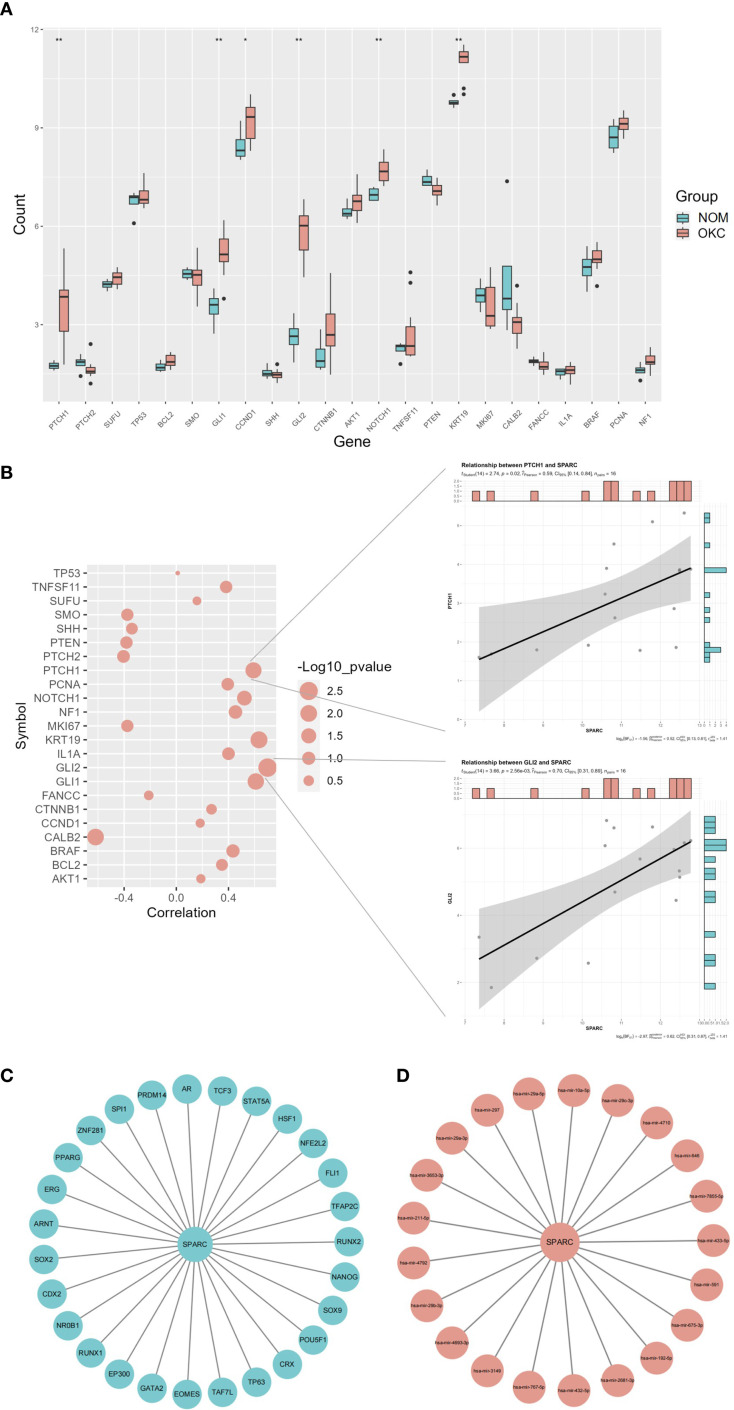
Correlation and regulatory network analysis of SPARC. **(A)** Differential expression of OKC-related genes in NOM and OKC samples. Blank indicates not significant; *P < 0.05; **P < 0.01. **(B)** Correlation analysis between SPARC and OKC-related genes (larger circles indicate lower P-values). Scatter plots on the right display correlations between SPARC and PTCH1, and between SPARC and GLI2. **(C)** TF and **(D)** miRNA regulatory network of SPARC. miRNA, microRNA; NOM, normal oral mucosa; OKC, odontogenic keratocyst; TF, transcription factor.

The top 23 genes associated with OKC were obtained from the GeneCards database, ranked by correlation scores. A boxplot was generated to visualize the differential expression of these genes across OKC and NOM samples (**Figure 4A**, **Supplementary Table S9**). SPARC was selected for further analysis, and pairwise correlation scatterplots were constructed between SPARC and each of the 23 OKC-related genes (**Figure 4B**, **Supplementary Table S10**). Among them, PTCH1, GLI1, GLI2, and KRT19 showed significant expression differences and were positively correlated with SPARC (P < 0.05).

Based on SPARC expression levels, the OKC samples were stratified into high and low expression groups, and GSVA was performed (**Supplementary Figure S4**, **Supplementary Table S11**). High SPARC expression was associated with biological processes such as “negative regulation of epithelial cell apoptosis process”, “axonogenesis involved in innervation”, and “positive regulation of response to type II interferon”, as well as molecular functions including “peptide alpha-N acetyltransferase activity”.

### 3.4 TF and miRNA regulatory network of SPARC

The TF and miRNA regulatory network for SPARC were predicted using NetworkAnalyst (**Figures 4C, D**). The TF network included 28 nodes and 27 edges, featuring pluripotency factors (POU5F1, SOX2, NANOG), nuclear receptors (PPARG, AR), and stress-responsive TFs (STAT5A, HSF1). Key hub nodes such as EP300 and RUNX1 suggested epigenetic and microenvironmental regulatory roles. The miRNA network included 22 nodes and 27 edges. Notable miRNAs included the miR-29 family (hsa-mir-29a-5p/3p) and cancer-associated miRNAs (hsa-mir-192-5p, hsa-mir-26b-3p). miR-29a-5p was identified as the highest-degree node, consistent with its known role in suppressing SPARC in fibrosis and metastasis.

### 3.5 SPARC is highly expressed in OKC fibroblasts and validated in independent datasets

**Figure 5:**
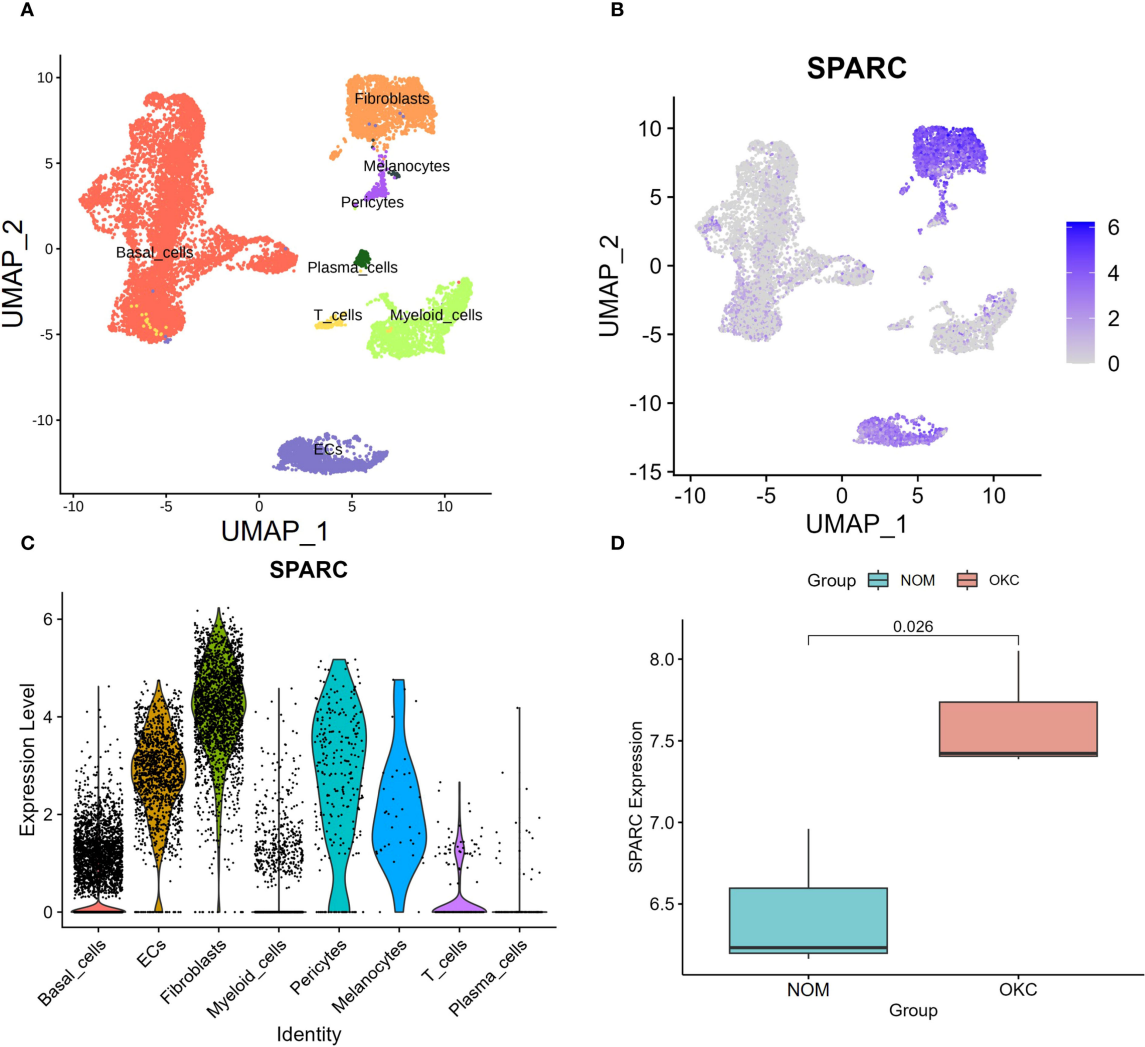
Validation of SPARC expression using single-cell and bulk RNA sequencing. **(A)** UMAP-based cell annotation and clustering for dataset GSE176351. Adapted from Man et al. (22), under CC BY 4.0 license. **(B)** Feature plot and **(C)** violin plot illustrating SPARC expression across cell populations. **(D)** Box plot validation of SPARC expression in NOM and OKC based on datasets GSE228393 and GSE186489 (P = 0.026). NOM, normal oral mucosa; OKC, odontogenic keratocyst.

ScRNA-seq data from three OKC samples (GSE176351) were merged and analyzed. UMAP visualization with annotated clusters is shown in **Figure 5A** (22). Feature plots (**Figure 5B**) and violin plots (**Figure 5C**) indicated that SPARC expression was highest in fibroblasts, followed by epithelial cells, and was present to a lesser extent in other cell types.

Two additional datasets (GSE228393 and GSE186489) were used to validate SPARC expression. After merging and batch effect removal using the “sva” package, a validation dataset was generated. Boxplots showed significantly higher SPARC expression in OKC compared to NOM (P = 0.026) (**Figure 5D**, **Supplementary Table S12**), confirming the expression pattern observed in the primary dataset.

### 3.6 SPARC is enriched in the stromal tissue of OKC compared to NOM

**Figure 6:**
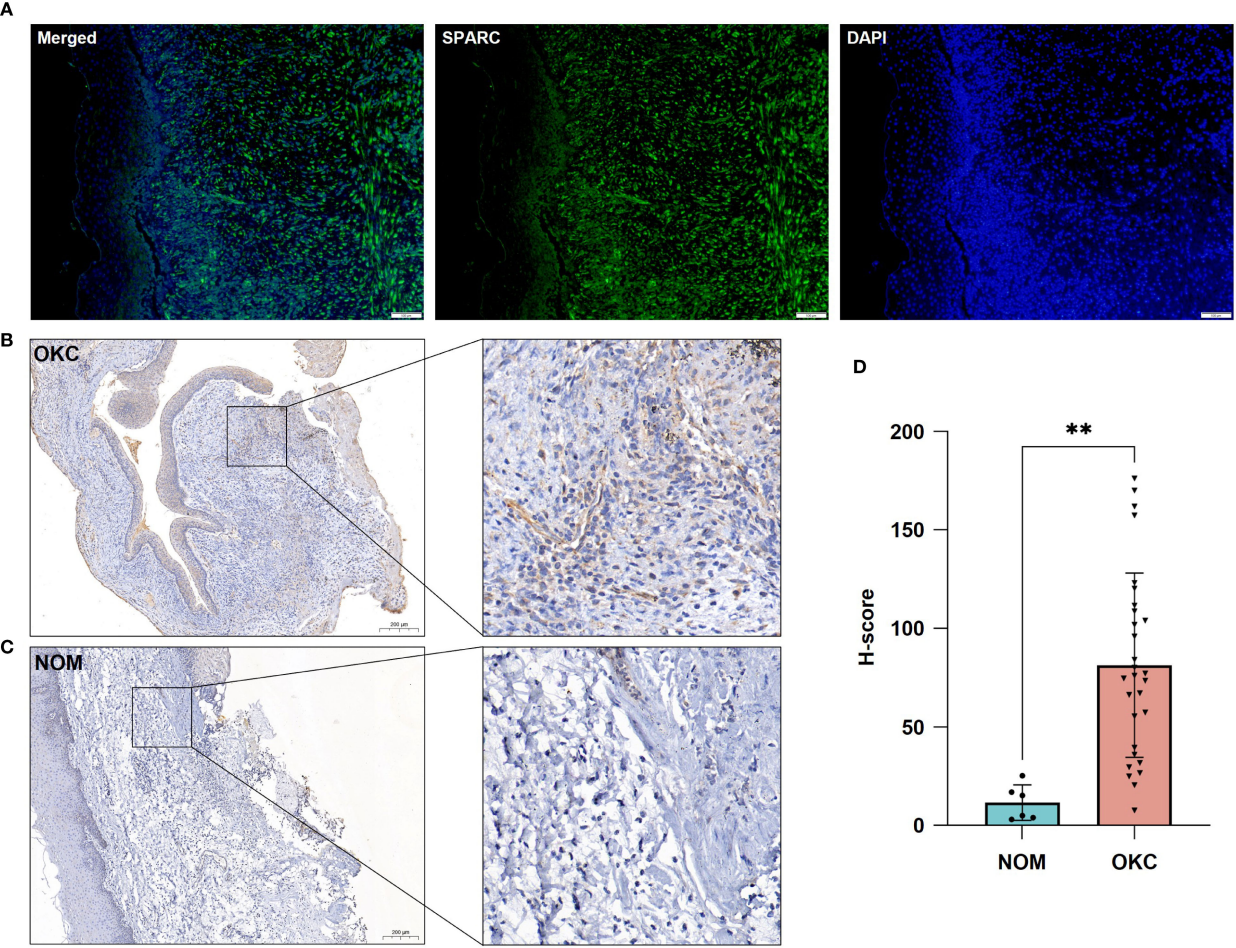
Validation of SPARC expression in OKC and NOM tissues. **(A)** Immunofluorescence showing SPARC expression in OKC tissue. Immunohistochemical staining for SPARC in **(B)** OKC and **(C)** NOM tissues. **(D)** Comparative analysis of SPARC expression between OKC and NOM tissues. **P < 0.01. NOM, normal oral mucosa; OKC, odontogenic keratocyst.

Immunofluorescence staining revealed strong SPARC expression in the stromal compartment of OKC tissue (**Figure 6A**). To compare SPARC expression between OKC and OM, immunohistochemical staining was performed on OKC (n = 29) and NOM (n = 6) samples (**Figures 6B, C**). SPARC was detected in both epithelium and stroma of OKC tissues, with predominant stromal expression. In NOM tissue, SPARC expression was relatively low. Quantitative analysis based on H-score demonstrated significantly higher SPARC expression in the stroma of OKC compared to NOM (P = 0.001) (**Figure 6D**).

### 3.7 SPARC expression correlates with infiltration of multiple immune cell types

**Figure 7:**
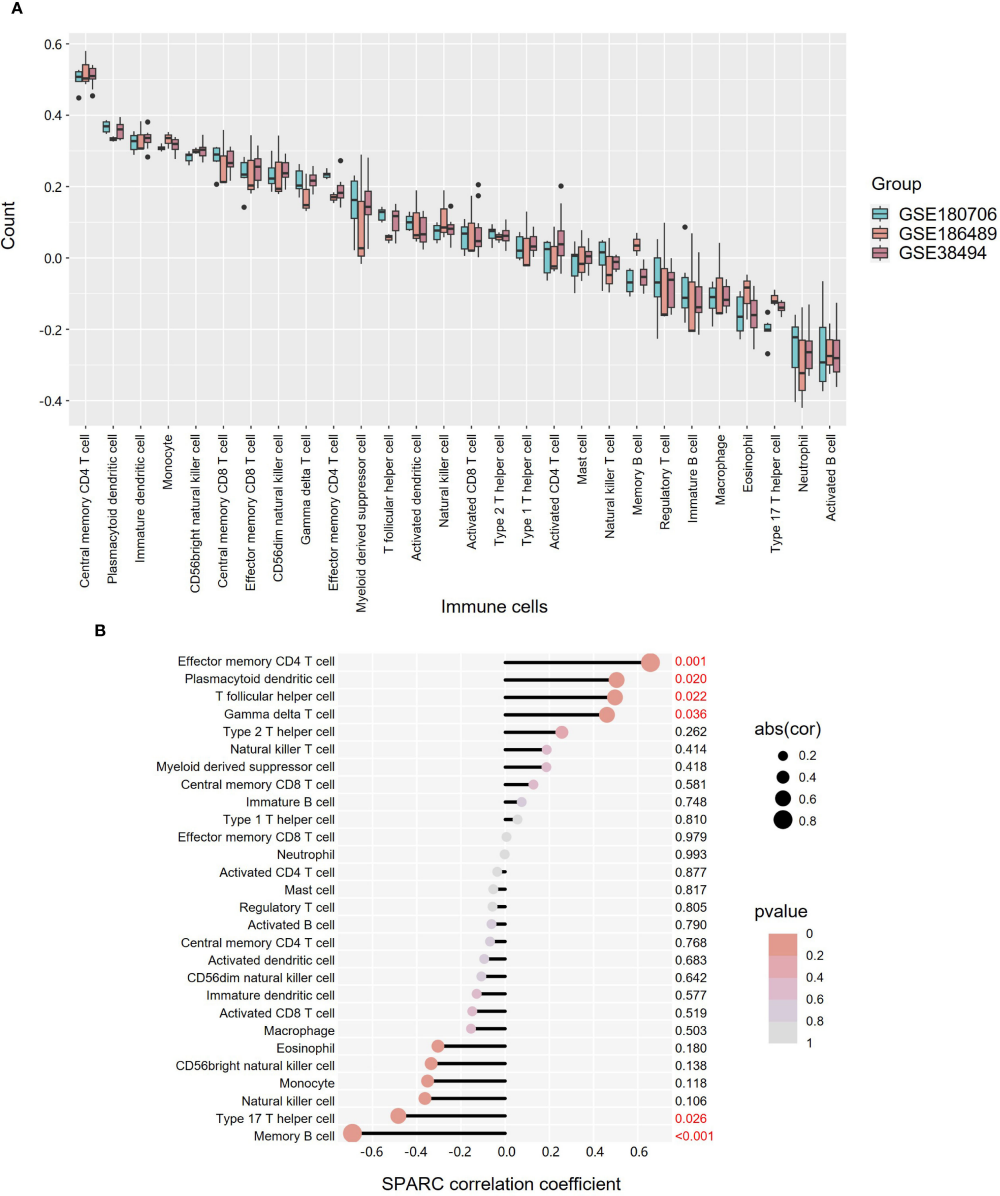
Expression and correlation analysis of 28 TIICs in OKC tissues. **(A)** Box plot showing the expression of 28 TIICs across datasets GSE180706, GSE186489, and GSE38494. **(B)** Correlation between TIICs and SPARC expression. Dot size indicates correlation strength, while color denotes P-value significance. OKC, odontogenic keratocyst; TIICs, tumor-infiltrating immune cells.

Immune infiltration analysis was conducted using gene sets for 28 TIIC types across three datasets (**Figure 7A**, **Supplementary Table S13**). The distribution of TIICs was broadly consistent across datasets. Correlation analysis between SPARC expression and TIICs showed that effector memory CD4^+^ T cells had the strongest positive correlation (P = 0.001), followed by plasmacytoid derivative cells (P = 0.020), T follicular helper cells (P = 0.022), and gamma delta T cells (P = 0.036). In contrast, memory B cells (P < 0.001) and Th17 cells *(*P = 0.026) negatively correlated with SPARC expression (**Figure 7B**, **Supplementary Table S14**).

## 4 Discussion

In this study, we identified 65 BM DEGs between OKC and OM samples through RNA-seq analysis and GEO database mining. Among them, SPARC emerged as a key gene and was further investigated through multiple bioinformatics approaches to clarify its potential localization and role in OKC progression.

PPI and gene correlation analyses revealed intricate interrelationships among the 65 BMGs, suggesting a tightly regulated BMGs network in OKC. GO enrichment analysis highlighted not only canonical ECM pathways but also biological processes such as “cell-substrate adhesion” and molecular functions like “integrin binding”. Previous studies have linked decreased expression of adhesion molecules, such as E-cadherin and α6β4 integrin, to increased tumor aggressiveness (23), supporting the relevance of these pathways in OKC. Consistent with this, KEGG analysis revealed significant enrichment in the “ECM–receptor interaction” and “focal adhesion” pathways.

SPARC, the most upregulated BM DEG in OKC, encodes a secreted matricellular protein—also known as osteonectin—that modulates ECM–cell interactions, inhibits cell proliferation, and regulates growth factor signaling. Some studies have demonstrated that SPARC may function as an extracellular chaperone or as a collagen chaperone, with the latter affecting the formation of fibrils *in vitro* (24). Under physiological conditions, SPARC is primarily expressed in remodeling tissues such as bone, intestinal mucosa, and healing wounds (25). Aberrant SPARC expression has been implicated in cancer and fibrotic diseases. For instance, SPARC promotes melanoma cell invasion by inducing Snail and repressing E-cadherin (26) and contributes to pulmonary fibrosis by regulating ECM turnover (27). In our analysis, SPARC expression positively correlated with several OKC-related genes, including PTCH1, NOTCH1, KRT19, GLI1, and GLI2. PTCH1, a key gene in OKC, has been shown to regulate SPARC and influence bone metabolism (28). NOTCH1 is associated with hypoxia responses in OKC, while KRT19 may reflect epithelial proliferative activity. GLI1 and GLI2, core effectors of the Hedgehog pathway, are established markers that distinguish NS-OKC from S-OKC (29–31).

Understanding the transcriptional and post-transcriptional regulation of SPARC is crucial to elucidating its role in both physiological and pathological contexts (32). In OKC, SPARC may be modulated by key transcription factors involved in epigenetic remodeling (e.g., EP300), epithelial plasticity (e.g., SOX2, NANOG), and ECM regulation (**Figures 4C, D**). MicroRNAs, particularly members of the miR-29 family, also regulate SPARC by suppressing its mRNA translation (33). For example, inhibition of miR-29a can restore SPARC expression and enhance ECM production (34), while miR-203 suppresses SPARC-driven metastasis in head and neck cancers (35). Additionally, SPARC has been shown to promote the expression of interferon-stimulated genes via IRF3/7 activation, contributing to the pro-inflammatory transformation of macrophages in aging tissues (36).

Our findings confirmed that SPARC expression is significantly elevated in the stromal compartment of OKC compared to NOM (P = 0.001), as evidenced by immunohistochemistry (**Figures 5B–D**). This observation is consistent with prior studies by Poomsawat et al. (37) and Hong et al. (28), who reported stromal SPARC expression in both S-OKC and NS-OKC. ScRNA-seq and immunofluorescence further validated that SPARC is predominantly expressed in OKC fibroblasts (**Figures 4A**, **5A**). A similar stromal enrichment of SPARC has been documented in pancreatic cancer, where it is associated with poor prognosis (38). As primary producers of SPARC, fibroblasts may contribute to lesion progression by promoting epithelial proliferation, osteoclastogenesis (39), and angiogenesis via LOXL4 activation (40), thereby enhancing the invasive behavior of OKC.

Our previous mass cytometry (CyTOF) analysis demonstrated that T cells, macrophages, neutrophils, and B cells dominate the immune microenvironment in OKC (41). In the current analysis, SPARC expression was negatively correlated with memory B cells, which not only participate in anti-tumor immunity through antibody production and T cell activation (42) but also secrete RANKL, promoting bone resorption and local inflammation (43). In SPARC-deficient mice, reductions in B cell numbers and lipopolysaccharide-induced immune responses have been observed (44), suggesting a potential regulatory role for SPARC in B cell development and function.

To further substantiate these findings, future studies could employ immunofluorescence double-labeling to directly visualize SPARC expression in specific leukocyte subsets, thereby providing confirmatory evidence for the scRNA-seq results. Such assays would help clarify whether stromal SPARC interacts with distinct immune populations in shaping the OKC microenvironment.

Nevertheless, this study has several limitations. Some are inherent to the research field rather than to this study itself. For instance, the limited availability of transcriptomic datasets for OKC in public repositories may restrict the robustness of bulk and single-cell RNA-seq analyses. In addition, technical challenges in isolating primary cells from OKC tissues and the absence of a reliable animal model constrain functional validation both *in vitro* and *in vivo*. At the level of the present work, the relatively small sample size and reliance on public databases may also introduce potential bias. Despite these limitations, our work provides important preliminary insights and lays a valuable foundation for future mechanistic investigations and therapeutic development targeting OKC.

In summary, this study systematically characterized BMG expression in OKC and identified dysregulation of adhesion-associated signaling pathways as potential contributors to lesion progression. Among these, SPARC emerged as a central regulatory molecule. Through integrated bioinformatics analyses—including co-expression profiling, transcription factor and miRNA network prediction, and immune cell infiltration mapping—we constructed a comprehensive regulatory network centered on SPARC. Our findings were further validated by scRNA-seq and histological assays, confirming elevated SPARC expression in the stromal compartment of OKC. Given SPARC’s pivotal role in the stromal microenvironment of OKC, targeting SPARC could potentially inhibit its involvement in lesion recurrence and stromal remodeling. Future clinical applications may focus on developing SPARC-based therapeutic strategies to prevent OKC progression and recurrence, offering a novel approach to managing this challenging disease.

## Data Availability

Publicly available datasets were analyzed in this study. This data can be found here: Gene Expression Omnibus (GEO) repository (bulk RNA-seq datasets: GSE38494, GSE180706, GSE186489; scRNA-seq dataset: GSE176351).
