# Supplementary material for: Function value of basement membrane-related genes in odontogenic keratocyst by bioinformatics analysis
Source: Front Oncol. 2025 Sep 12;15:1658125. doi: 10.3389/fonc.2025.1658125 (PMC12463645; doi:10.3389/fonc.2025.1658125)
Supplement: Supplementary file 1 [file DataSheet1.zip › Supplementary Figures.docx]

Supplementary Figures


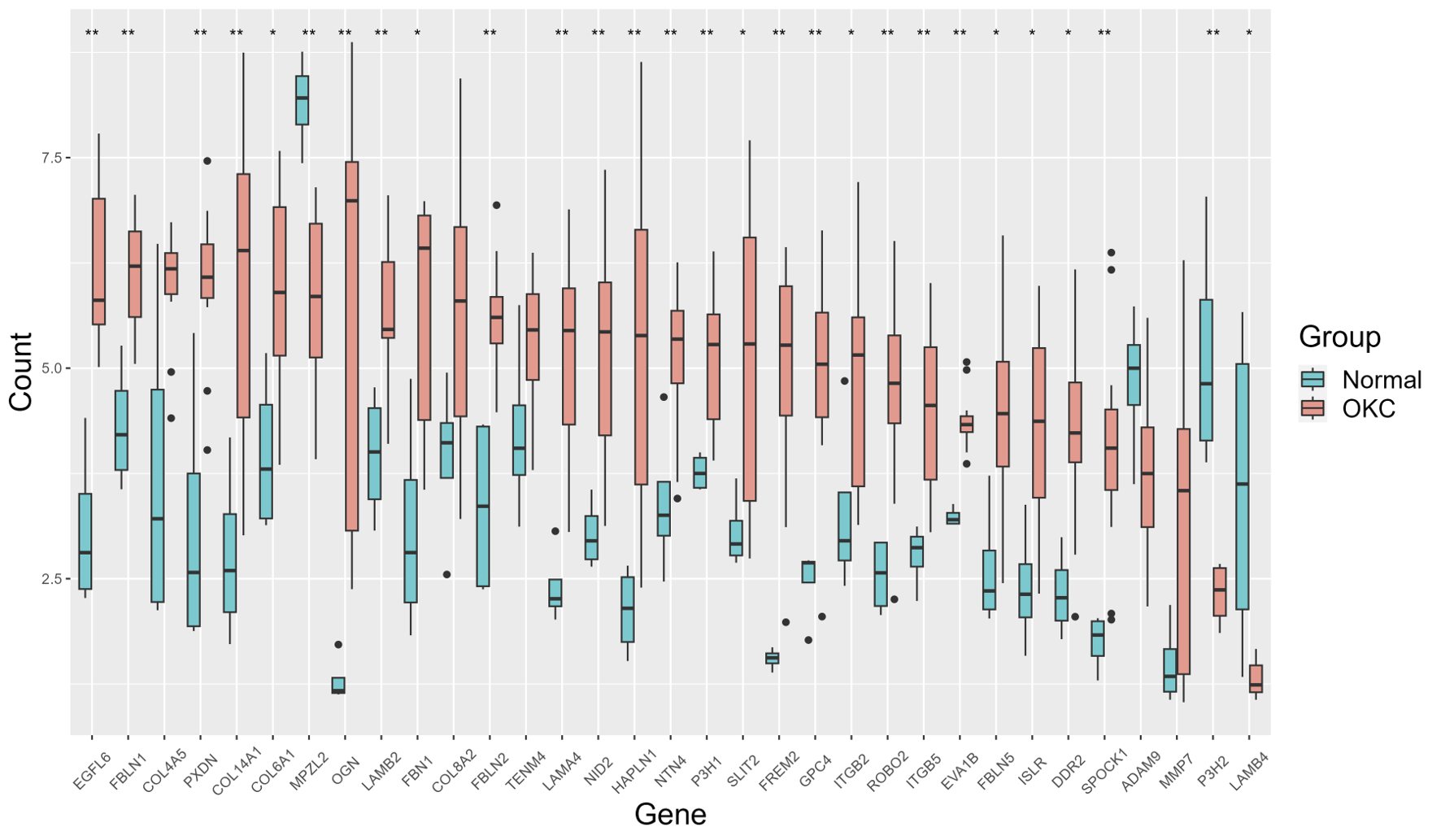


**Figure S1. Differential expression analysis of the remaining 33 BM-DEGs in OKC.** Blank, not significant, **P* < 0.05, ***P* < 0.01. OKC: odontogenic keratocyst, BM: basement membrane, DEGs: differentially expressed genes.


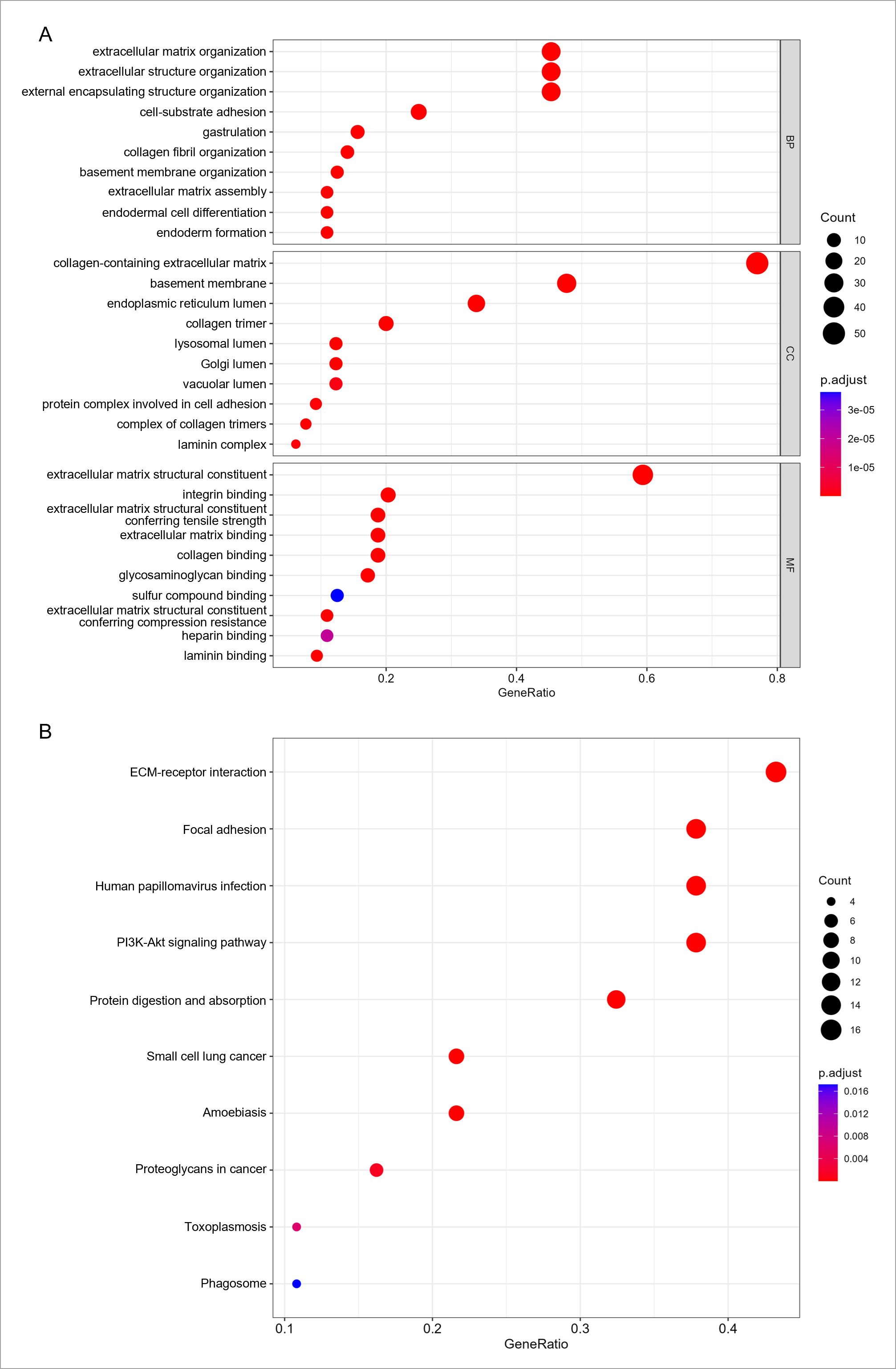


**Figure S2. Functional enrichment analysis of BM-DEGs.** (A) GO enrichment analysis of the BM-DEGs. (B) KEGG pathway enrichment analysis of the BM-DEGs. GO: Gene Ontology, KEGG: Kyoto Encyclopedia of Genes and Genomes, BM: basement membrane, DEGs: differentially expressed genes.


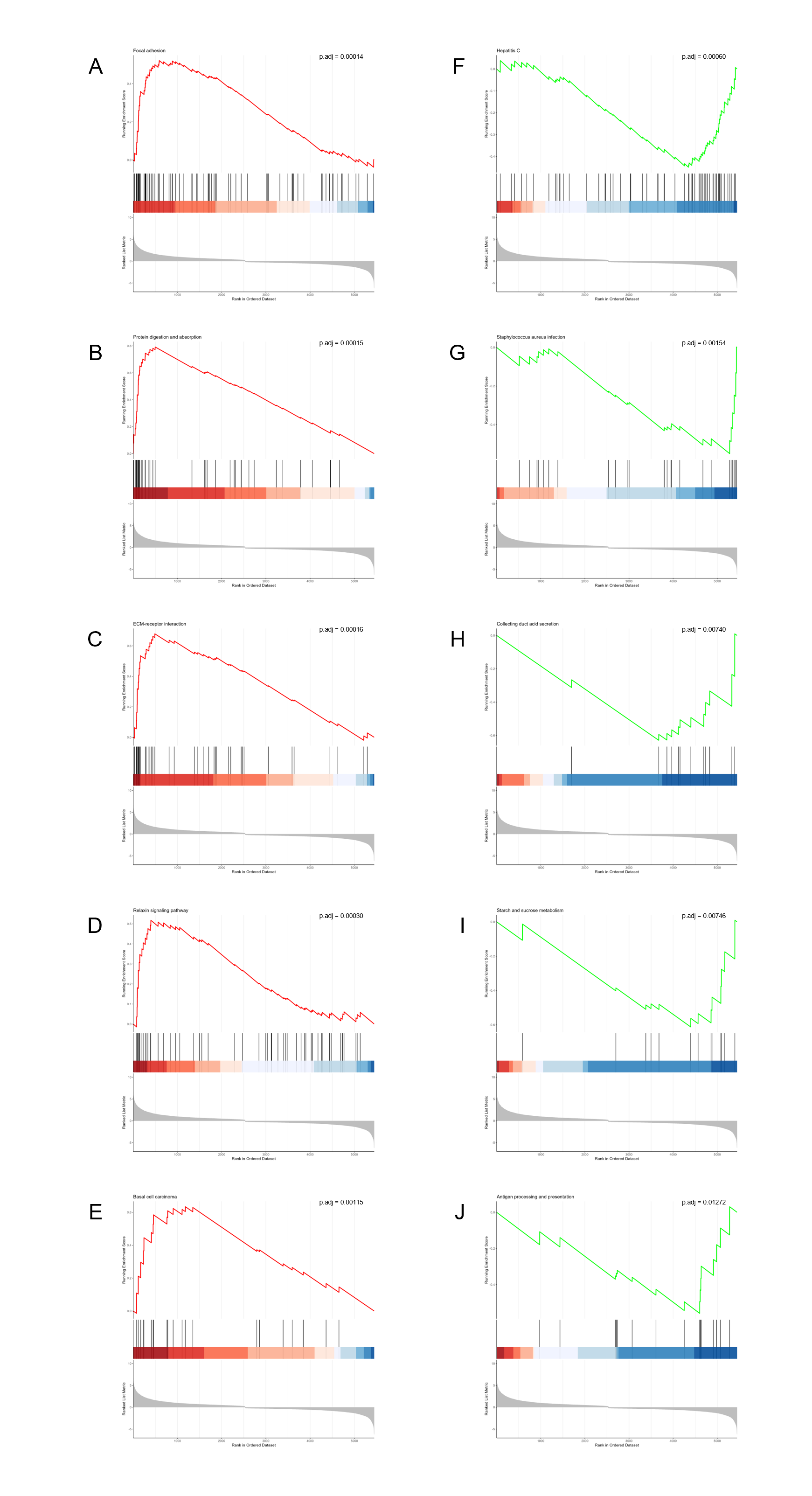


**Figure S3. GSEA of BM-DEGs.** The figure showcases the top five upregulated (A-E) and top five downregulated (F-J) pathways among all DEGs in OKC and OM samples. GSVA: gene set enrichment analysis, BM: basement membrane, DEGs: differentially expressed genes, OKC: odontogenic keratocyst, OM: oral mucosa.


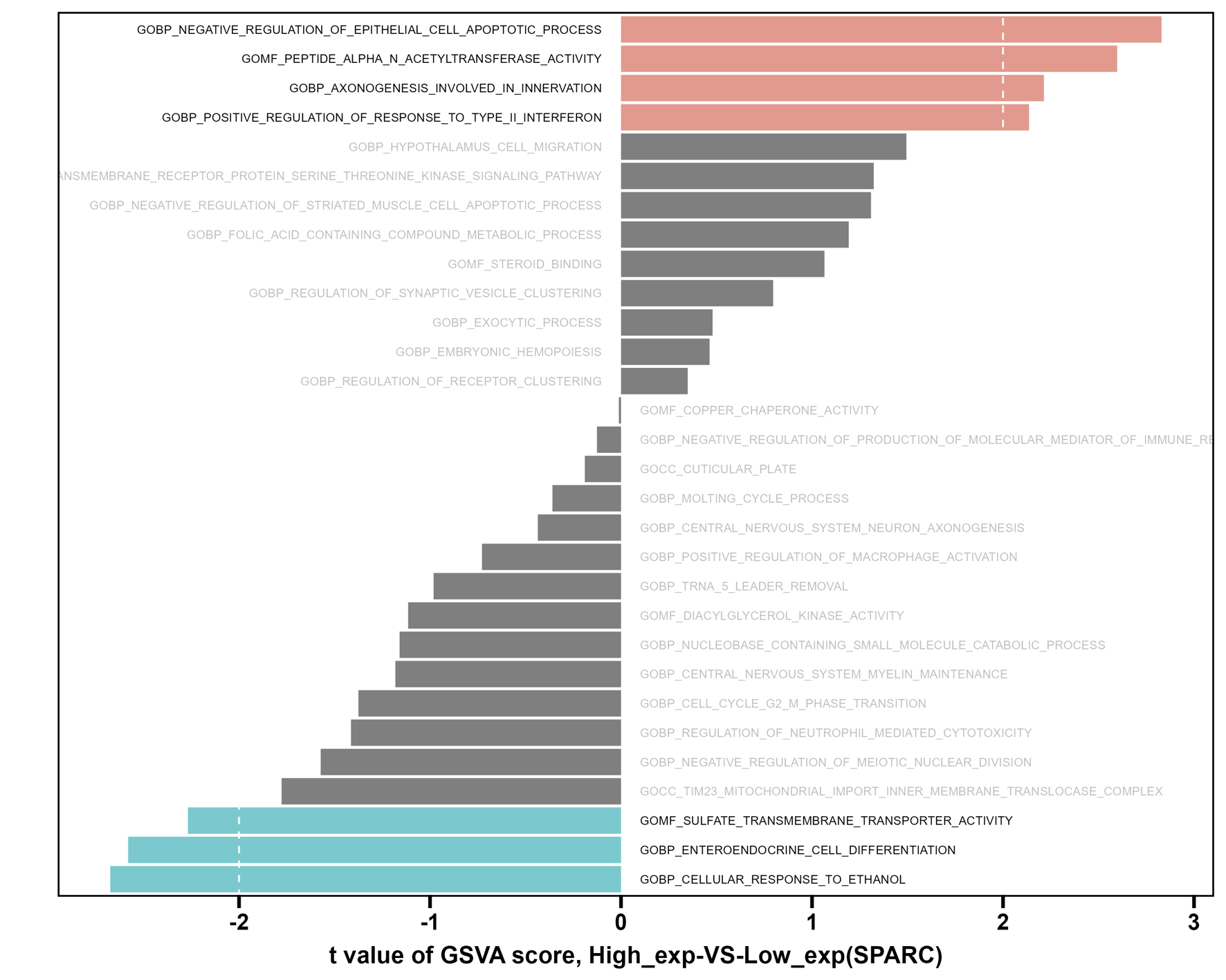


**Figure S4. GSVA comparing high and low SPARC expression groups in OKC samples, based on gene sets in the GO database.** GO: Gene Ontology, GOBP: GO biological process, GOMF: GO molecular function, GSVA: gene set variation analysis, OKC: odontogenic keratocyst.
